# The prevalence of chronic conditions in patients diagnosed with one of 29 common and rarer cancers: A cross-sectional study using primary care data

**DOI:** 10.1016/j.canep.2020.101845

**Published:** 2020-12

**Authors:** Minjoung Monica Koo, Ruth Swann, Sean McPhail, Gary A. Abel, Cristina Renzi, Greg P. Rubin, Georgios Lyratzopoulos

**Affiliations:** a University College London, 1-19 Torrington Place, London WC1E 6BT, UK; b National Cancer Registration and Analysis Service, Public Health England, Wellington House, 133-155 Waterloo Road, London, SE1 8UG, UK; c Cancer Research UK, 2 Redman Place, London, E20 1JQ, UK; d University of Exeter Medical School, St Luke's Campus, Heavitree Road, Exeter, EX1 2LU, UK; e Institute of Health and Society, Newcastle University, Sir James Spence Institute, Royal Victoria Infirmary, Newcastle upon Tyne, NE1 4LP, UK

**Keywords:** Comorbidities, Long-term conditions, Oncology, Cancer outcomes, Epidemiology

## Abstract

**Background:**

Pre-existing chronic conditions (morbidities) influence the diagnosis and management of cancer. The prevalence of specific morbidities in patients diagnosed with common and rarer cancers is inadequately described.

**Methods:**

Using data from the English National Cancer Diagnosis Audit 2014, we studied 11 pre-existing morbidities recorded as yes/no items by participating general practitioners based on information included in primary care records. We examined the number and type of morbidities across socio-demographic and cancer site strata, and subsequently estimated observed and age/sex standardised prevalence of each morbidity by cancer.

**Results:**

Over three-quarters (77 %; 11,429/14,774) of non-screen-detected patients had at least one chronic condition before diagnosis, while nearly half (47 %) had two or more. Hypertension (39 %) and physical disability (2%) were the most and least common conditions. Male, older and more socio-economically deprived patients were more likely to have at least one morbidity (p < 0.001 for all between variable group comparisons). For most morbidities, the standardised prevalence was similar across different cancers with a few exceptions, including respiratory disease prevalence being greatest among lung cancer patients and diabetes prevalence being greatest among liver, pancreatic, and endometrial cancer patients.

**Conclusions:**

Most cancer patients have at least one morbidity, while almost one in two have two or more. The findings highlight the need to take certain morbidity- and cancer-site combinations into account when examining associations between morbidity and cancer outcomes.

## Highlights

• Most patients had at least one morbidity; nearly one in two had multiple morbidities.
• Morbidity prevalence was similar across cancers but with notable exceptions.
• Related enquiries should take account of certain combinations of morbidities and cancer.

## 1 Background

Population ageing is contributing to the rising prevalence of chronic conditions (morbidities) and increasing cancer incidence [[1], [2], [3], [4], [5], [6]]. However, for most cancers, detailed appreciation of their morbidity profile is lacking.

Understanding the burden and type of pre-existing morbidities is important when examining variation in diagnosis and management of a new cancer. Comorbidities may share common risk factors with different cancers, and influence healthcare utilisation pathways to the diagnosis of cancer and decisions about its treatment [7,8]. Recent research examining morbidity and cancer in light of the COVID-19 pandemic has highlighted a substantial excess mortality burden in patients with multiple conditions [9].

Prior studies have described the prevalence of morbidity among cancer patients using information from hospital records [[10], [11], [12]]. By their design, such studies under-estimate the prevalence of conditions that are principally managed in primary care, as these typically do not require hospital admissions [13,14]. Other studies using primary care data typically focus on single common cancer sites such as colorectal cancer [[15], [16], [17], [18]].

In this study, we aimed to use primary care derived data to describe the prevalence and type of pre-existing conditions among a representative cohort of incident cancer cases identified from a population-based cancer registry, in order to better inform and target future research about the likely influence of morbidity on diagnostic investigations and treatment outcomes.

## 2 Methods

### 2.1 Data and study population

We examined data from the National Cancer Diagnosis Audit (NCDA) 2014, described in detail previously [19]. Briefly, Public Health England’s National Cancer Registration and Analysis Service (NCRAS) identified incident cancer cases diagnosed in 2014 in England. These cases were assigned to the general practice at which they were registered at the time of diagnosis, and then participating General Practitioners (GPs) or other primary care professionals provided information on the cancer patients’ diagnostic process based on their records [19].

**Fig. 1:**
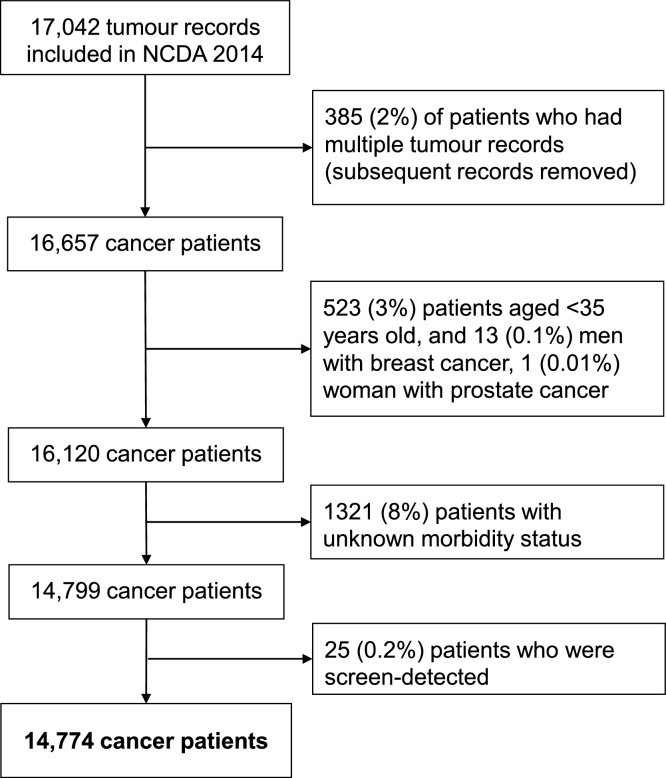
Flow chart indicating sample derivation.

Sample derivation is described in Fig. 1. After excluding subsequent records of individuals with multiple tumours, we excluded patients aged younger than 35 years at diagnosis; those with screen-detected cancers, as auditors were not required to submit information for such cases [19]; and those with missing morbidity information.

### 2.2 Variables of interest

Participating healthcare professionals provided information on patient characteristics prior to their cancer diagnosis based on their primary care records. This included information on whether any of the following morbidities were present: hypertension, cardiovascular disease (CVD), arthritis/musculo-skeletal disease (MSK disease), diabetes, chronic respiratory illness (respiratory disease), cerebrovascular disease (CBD), cognitive impairment, physical disability, previous cancer, or other [unspecified] comorbidity. Each patient could therefore have no morbidities, a single morbidity, or two or more of the 11 morbidity categories in any combination (multimorbidity).

Additionally, patient-level information from cancer registration was extracted on sex (male or female); age group (35–44 years, 45–54 years, 55–64 years, 65–74 years, 75–84 years, and 85+ years); ethnicity (white, non-white, and unknown); socio-economic deprivation group (quintiles of Index of Multiple Deprivation (IMD) income domain scores, where 1 indicated least deprived and 5 indicated most deprived); and cancer site based on ICD-10 codes (29 sites, which ordered by decreasing sample size were: prostate, lung, breast, colon, melanoma, lymphoma, other, rectal, renal, bladder, pancreatic, oesophageal, leukaemia, endometrial, cancer of the unknown primary (CUP), ovarian, stomach, oral/oropharyngeal, myeloma, liver, brain/CNS, mesothelioma, thyroid, laryngeal, small intestine, cervical, testicular, vulval, and gallbladder).

### 2.3 Statistical analysis

Briefly, our aim was to describe the overall distribution of morbidities among the cancer patient population and associations between morbidity and patient characteristics; and to examine the prevalence of specific morbidities across different cancer sites.

Firstly, we described the median, inter-quartile range, and proportion of patients with 0, 1, 2, 3, and 4+ morbidities, by patient characteristics (sex, age group, ethnicity, IMD income domain quintiles, and subsequently diagnosed cancer site). To further examine patient-level factors associated with morbidity we used logistic regression, treating the presence of at least one morbidity as the binary outcome of interest (vs no morbidity). Joint Wald tests were used to assess statistical significance of differences in morbidity prevalence across categorical variables. In regression analyses, reference groups were: males, 65–74 years, white ethnicity, least socio-economically deprived quintile group, and colon cancer (chosen as a common cancer that is not sex-specific).

We then estimated the observed (crude) and directly standardised prevalence of each of the 11 specified morbidities among the study population. Specifically, we calculated the observed prevalence of each of the 11 specified morbidities by cancer site for 21 cancers with a sample size of at least 200 patients (excluding mesothelioma, thyroid, laryngeal, small intestine, cervical, testicular, vulval, and gallbladder cancers). The observed prevalence of morbidities by cancer is influenced by case-mix differences between cancer groups (e.g. women with breast cancer tend to be younger than those diagnosed with most other cancer types, and so typically have lower prevalence of morbidities prior to cancer diagnosis). For this reason we then directly standardised the prevalence of morbidities using corresponding age and sex stratum-specific mid-year (2014) English population estimates [20]. This was conducted using Stata’s *proportion* command with the *stdize* option. There were no men with bladder cancer aged 35−44 years in the study population; standardised prevalence estimates of morbidity among bladder cancer patients are therefore based on a population excluding this age- and sex-specific stratum. Supplementary Material describes the age and sex structure of the standard population and compares it to that of our study (Supplementary Material I).

All analyses were conducted using Stata SE version 15.1 (StataCorp, College Station, TX, USA; 2017).

## 3 Results

### 3.1 Number of morbidities and patient characteristics

Of the 14,774 cancer patients in our study population, 7883 (53 %) were male, mean age of 70 years and median (IQR) age of 71 (61–79) years, and 88 % were white (Table 1).

**Table 1:** Study population, and median (inter-quartile range (IQR)) of number of morbidities by patient characteristic; and proportion of patients with at least one morbidity, multi-morbidity, and crude/adjusted odds ratios of at least one morbidity by patient characteristic (n = 14,774).

	N (% of total)	Median (IQR) n. of comorbidities	At least one morbidity, n(%)	Multi-morbidity (two or more morbidities), n(%)	Crude OR for at least one morbidity (95 % CI)	Joint Wald test	Adjusted OR for at least one morbidity (95 % CI)*	Joint Wald test
Total	14,774 (100 %)	1 (1–2)	11,429 (77 %)	6898 (47 %)	–		–	
Sex								
Male	7883 (53 %)	1 (1–2)	6285 (80 %)	3840 (49 %)	Ref		Ref	
Female	6891 (47 %)	1 (0–2)	5144 (75 %)	3058 (44 %)	0.75 (0.69–0.81)	<0.001	0.80 (0.72–0.89)	<0.001
Age group								
35−44 years	609 (4 %)	0 (0–1)	196 (32 %)	40 (7%)	0.11 (0.09–0.13)		0.12 (0.10–0.15)	
45–54 years	1449 (10 %)	0 (0–1)	672 (46 %)	202 (14 %)	0.20 (0.18–0.23)		0.21 (0.18–0.24)	
55–64 years	2630 (18 %)	1 (0–2)	1752 (67 %)	821 (31 %)	0.46 (0.41–0.52)		0.46 (0.41–0.51)	<0.001
65–74 years	4268 (29 %)	1 (1–2)	3464 (81 %)	2044 (48 %)	Ref		Ref	
75–84 years	3981 (27 %)	2 (1–3)	3629 (91 %)	2518 (63 %)	2.39 (2.09–2.74)		2.41 (2.10–2.75)	
85+ years	1837 (12 %)	2 (1–3)	1716 (93 %)	1273 (69 %)	3.29 (2.70–4.02)		3.35 (2.74–4.10)	
Ethnicity								
White	12,940 (88 %)	1 (1–2)	10,060 (78 %)	6111 (47 %)	Ref		Ref	
Non-white	616 (4 %)	1 (0–2)	452 (73 %)	259 (42 %)	0.79 (0.66–0.95)	0.008	1.17 (0.95–1.44)	0.249
Missing	1218 (8 %)	1 (1–2)	917 (75 %)	528 (43 %)	0.87 (0.76–1.00)		0.95 (0.82–1.11)	
IMD quintile								
1 - least deprived	3153 (21 %)	1 (0–2)	2314 (73 %)	1296 (41 %)	Ref		Ref	
2	3269 (22 %)	1 (1–2)	2481 (76 %)	1426 (44 %)	1.14 (1.02–1.28)		1.10 (0.97–1.24)	
3	3204 (22 %)	1 (1–2)	2510 (78 %)	1531 (48 %)	1.31 (1.17–1.47)	<0.001	1.25 (1.10–1.43)	<0.001
4	2746 (19 %)	1 (1–2)	2159 (79 %)	1340 (49 %)	1.33 (1.18–1.50)		1.39 (1.22–1.60)	
5 - most deprived	2402 (16 %)	2 (1–3)	1965 (82 %)	1305 (54 %)	1.63 (1.43–1.86)		1.73 (1.50–2.00)	
Cancer site								
Gallbladder	48 (0.3 %)	2 (1–2)	45 (94 %)	31 (65 %)	3.66 (1.13–11.88)		3.75 (1.12–12.62)	
Liver	256 (2 %)	2 (1–3)	230 (90 %)	161 (63 %)	2.16 (1.40–3.32)		2.46 (1.56–3.88)	
Lung	2037 (14 %)	2 (1–3)	1767 (87 %)	1200 (59 %)	1.60 (1.31–1.94)		1.49 (1.21–1.84)	
Bladder	467 (3 %)	2 (1–3)	405 (87 %)	244 (52 %)	1.59 (1.18–2.16)		1.24 (0.89–1.71)	
Laryngeal	99 (1 %)	1 (1–2)	85 (86 %)	43 (43 %)	1.48 (0.83–2.66)		1.85 (1.00–3.40)	
Myeloma	262 (2 %)	1 (1–2)	223 (85 %)	126 (48 %)	1.40 (0.96–2.02)		1.53 (1.03–2.28)	
CUP	381 (3 %)	2 (1–3)	322 (85 %)	214 (56 %)	1.33 (0.97–1.82)		1.19 (0.85–1.67)	
Mesothelioma	145 (1 %)	1 (1–2)	121 (83 %)	72 (50 %)	1.23 (0.78–1.95)		0.94 (0.58–1.53)	
Renal	520 (4 %)	2 (1–3)	428 (82 %)	268 (52 %)	1.14 (0.87–1.48)		1.56 (1.17–2.09)	
Pancreatic	446 (3 %)	2 (1–2)	367 (82 %)	227 (51 %)	1.13 (0.85–1.51)		1.12 (0.82–1.51)	
Oesophageal	427 (3 %)	1 (1–3)	350 (82 %)	208 (49 %)	1.11 (0.83–1.48)		1.05 (0.77–1.43)	
Colon	1137 (8 %)	2 (1–2)	914 (80 %)	572 (50 %)	Ref	<0.001	Ref	<0.001
Stomach	295 (2 %)	2 (1–3)	237 (80 %)	153 (52 %)	1.00 (0.72–1.38)		0.95 (0.67–1.35)	
Leukaemia	398 (3 %)	1 (1–2)	312 (78 %)	165 (41 %)	0.89 (0.67–1.17)		1.09 (0.80–1.49)	
Vulval	53 (0.4 %)	2 (1–3)	41 (77 %)	27 (51 %)	0.83 (0.43–1.61)		1.29 (0.61–2.73)	
Prostate	2082 (14 %)	1 (1–2)	1605 (77 %)	927 (45 %)	0.82 (0.69–0.98)		0.78 (0.64–0.95)	
Lymphoma	653 (4 %)	1 (1–2)	498 (76 %)	290 (44 %)	0.78 (0.62–0.99)		1.05 (0.82–1.36)	
Other	609 (4 %)	1 (1–2)	459 (75 %)	274 (45 %)	0.75 (0.59–0.94)		1.05 (0.81–1.35)	
Endometrial	385 (3 %)	1 (0–2)	285 (74 %)	186 (48 %)	0.70 (0.53–0.91)		1.04 (0.78–1.41)	
Rectal	554 (4 %)	1 (0–2)	408 (74 %)	248 (45 %)	0.68 (0.54–0.87)		0.80 (0.61–1.03)	
Small Intestine	68 (0.5 %)	1 (0–2)	49 (72 %)	29 (43 %)	0.63 (0.36–1.09)		0.80 (0.44–1.45)	
Oral/oropharyngeal	291 (2 %)	1 (0–2)	206 (71 %)	107 (37 %)	0.59 (0.44–0.79)		0.93 (0.68–1.28)	
Ovarian	318 (2 %)	1 (0–2)	222 (70 %)	110 (35 %)	0.56 (0.43–0.75)		0.91 (0.66–1.24)	
Breast	1630 (11 %)	1 (0–2)	1088 (67 %)	597 (37 %)	0.49 (0.41–0.59)		0.96 (0.78–1.18)	
Melanoma	761 (5 %)	1 (0–2)	506 (66 %)	291 (38 %)	0.48 (0.39–0.60)		0.82 (0.65–1.03)	
Thyroid	113 (1 %)	1 (0–2)	72 (64 %)	31 (27 %)	0.43 (0.28–0.65)		1.28 (0.82–2.02)	
Brain/CNS	213 (1 %)	1 (0–2)	131 (62 %)	73 (34 %)	0.39 (0.29–0.53)		0.61 (0.43–0.86)	
Cervical	65 (0.4 %)	0 (0–2)	32 (49 %)	17 (26 %)	0.24 (0.14–0.39)		0.61 (0.34–1.09)	
Testicular	61 (0.4 %)	0 (0–1)	21 (34 %)	7 (11 %)	0.13 (0.07–0.22)		0.65 (0.36–1.17)	

CNS: central nervous system; CUP: cancer of unknown primary; IMD: index of multiple deprivation.

*adjusting for sex, age, ethnicity, IMD, and cancer site.

**Fig. 2:**
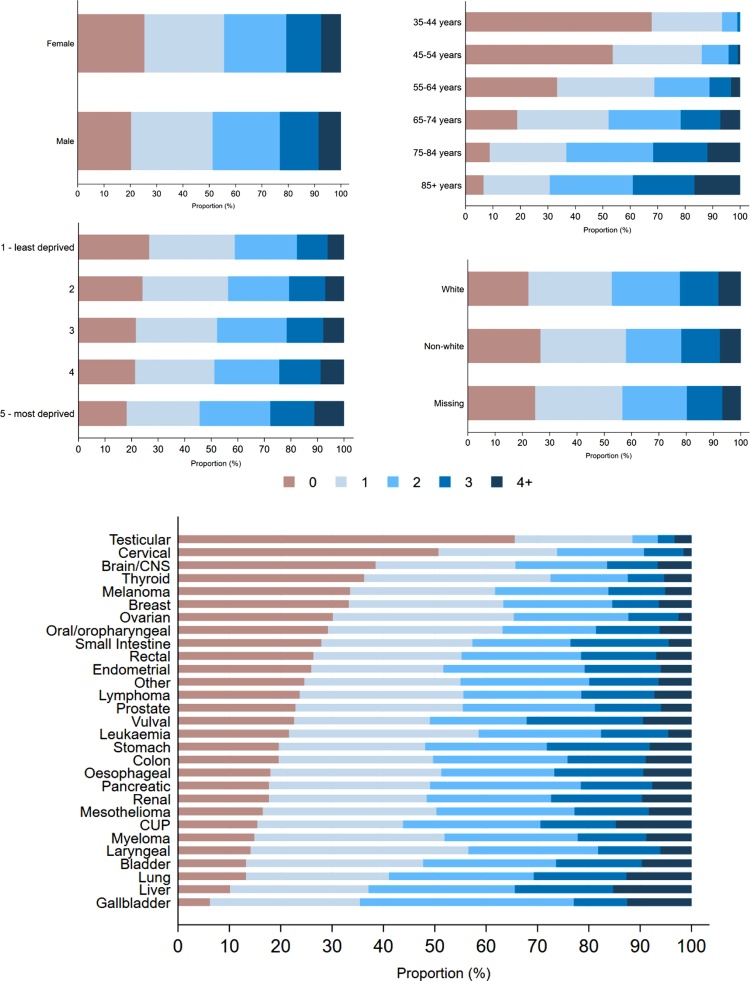
Number of comorbidities (0, 1, 2, 3, 4+) by sex, age group, ethnicity, income deprivation quintile, and cancer site. CNS: central nervous system; CUP: cancer of unknown primary.

More than three-quarters (77 %; 11,429/14,774) of patients had at least one recorded pre-existing condition and almost half (47 %) had two or more conditions before cancer diagnosis (Table 1 and Fig. 2). The prevalence of at least one morbidity varied greatly with increasing age (ranging from 32 % of 35–44 year olds to 93 % of 85+ year olds, p < 0.001), and was slightly greater among men (80 % of men vs 75 % of women) and those in more socio-economically deprived quintiles (ranging from 73 % to 82 %, p < 0.001 for both). For all but two of the 29 cancer sites, the majority (more than half) of patients had one or more morbidities before diagnosis (Table 1 and Fig. 2).

Multivariable logistic regression (adjusting for all socio-demographic variables and cancer site) indicated similar overall patterns of socio-demographic variation to those observed in crude analyses. The size of variation in the presence of morbidity by cancer site diminished substantially though remained large (decreasing from 28-fold to 6-fold variation in the range of crude or adjusted odds ratios, respectively).

### 3.2 Prevalence of individual chronic conditions and co-occurrence

Of the 11 examined morbidities, hypertension was the most common, (39 % of all patients, 5752/14,774), while physical disability was the least common, (2 %, 249/14,774) (Table 2).

**Table 2:** Observed and standardised prevalence of individual morbidities and their observed co-occurrence (n = 14,774).

				Total number of morbidities (observed co-occurrence)			
Morbidity	Total N	Observed prevalence % (95 % CI)	Standardised prevalence % (95 % CI)	As a single morbidity	One additional morbidity	Two additional morbidities	Three or more additional morbidities
Hypertension	5752	39 % (38–40 %)	24 % (24–25 %)	1276 (22 %)	1970 (34 %)	1491 (26 %)	1015 (18 %)
CVD	3144	21 % (21–22 %)	11 % (11–12 %)	471 (15 %)	913 (29 %)	951 (30 %)	809 (26 %)
Other morbidity	2972	20 % (19–21 %)	19 % (18–19 %)	818 (28 %)	951 (32 %)	684 (23 %)	519 (17 %)
MSK disease	2706	18 % (18–19 %)	12 % (12–13 %)	518 (19 %)	860 (32 %)	728 (27 %)	600 (22 %)
Diabetes	2389	16 % (16–17 %)	11 % (11–12 %)	308 (13 %)	766 (32 %)	713 (30 %)	602 (25 %)
Respiratory disease	2263	15 % (15–16 %)	11 % (11–12 %)	481 (21 %)	678 (30 %)	604 (27 %)	500 (22 %)
Previous cancer	1650	11 % (11–12 %)	7% (7–8%)	370 (22 %)	505 (31 %)	410 (25 %)	365 (22 %)
CBD	1056	7 % (7–8 %)	4% (3–4%)	73 (7%)	279 (26 %)	324 (31 %)	380 (36 %)
Cognitive impairment	671	5 % (4–5%)	2% (2–3%)	88 (13 %)	188 (28 %)	188 (28 %)	207 (31 %)
Severe mental illness	373	3 % (2–3 %)	3% (3–3%)	111 (30 %)	119 (32 %)	77 (21 %)	66 (18 %)
Physical disability	249	2 % (1–2 %)	1% (1–1%)	17 (7%)	53 (21 %)	55 (22 %)	124 (50 %)

CVD = cardiovascular disease; MSK disease = arthritis/musculo-skeletal disease; CBD = cerebrovascular disease.

Severe mental illness was most commonly reported as a single pre-existing condition (30 % of patients with this condition had no other morbidity). In comparison, the vast majority of those with cerebrovascular disease (CBD) or physical disability had other morbidities, most commonly hypertension, cardiovascular disease (CVD), and other [undefined] morbidity (only 7% of patients with either of these conditions did not have at least one other morbidity).

### 3.3 Observed and standardised morbidity prevalence by cancer site

**Fig. 3:**
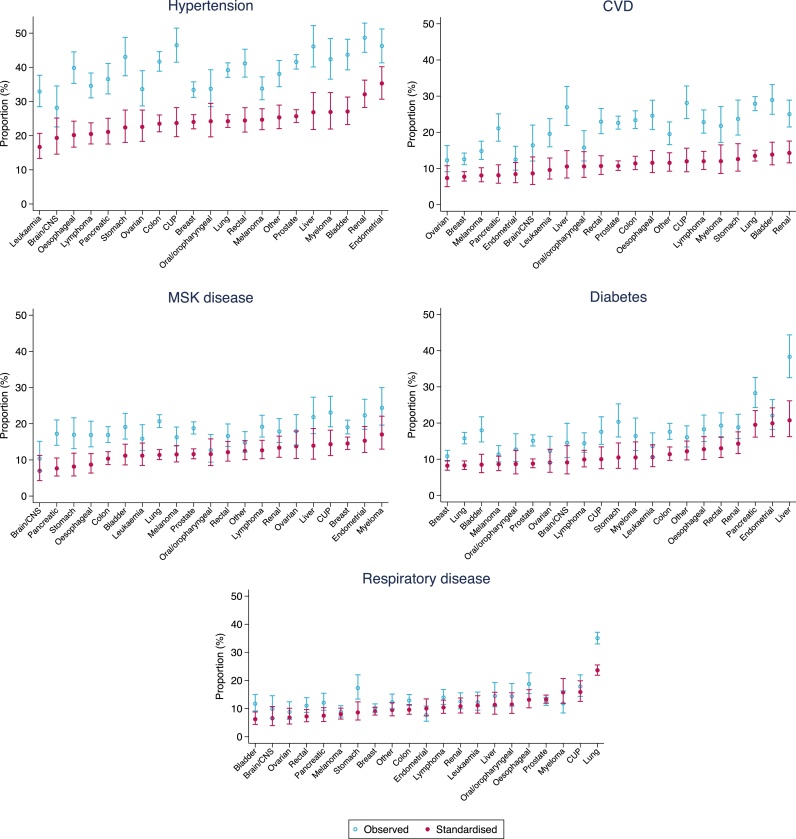
A–E Observed and directly standardised prevalence of morbidity by cancer site, for the five most frequent morbidities. Observed prevalence is indicated with hollow blue circles, while standardised prevalence is indicated with filled red circles. Cancer sites are ordered by increasing standardised prevalence. See Supplementary material II and III for underlying prevalence estimates (For interpretation of the references to colour in this figure legend, the reader is referred to the web version of this article).

Morbidity prevalence by cancer site (visualised by morbidity) is shown in Fig. 3A–E for the five most common morbidities excluding “other morbidity” (prevalence estimates for all 11 morbidities by cancer are presented in Supplementary material II and III). Standardised prevalence estimates were typically lower than the observed prevalence, given that cancer patients in our sample were on average older than the general population (see Supplementary material I).

For most morbidities, there was limited variation in standardised prevalence between different cancers as indicated by the overlap in standardised morbidity prevalence estimates by cancer and the 95 % confidence intervals. There were however a few notable exceptions to this pattern, for example the prevalence (95 % CI) of diabetes was relatively high among liver (21 % (16–26 %)), endometrial (20 % (16–24 %)), and pancreatic (20 % (16–23 %)) cancer patients (Fig. 3D) compared to overall prevalence of diabetes in the study population (16 % (16–17 %)). Similarly, respiratory disease was more common among lung cancer patients (24 % (22–26 %)) compared to overall prevalence of 15 % (15–16 %) (Fig. 3E). To help appreciate the morbidity burden for patient groups with different cancers, the same prevalence estimates are visualised by cancer site in the appendix (Supplmentary material IV).

## 4 Discussion

### 4.1 Summary of findings

More than three-quarters of cancer patients have one or more pre-existing conditions prior to cancer diagnosis. Prior chronic conditions are particularly prevalent among older patients, and also among socio-economically deprived patients and men. The standardised prevalence of the studied conditions was largely comparable among patients diagnosed with different cancers with a few exceptions such as higher prevalence of respiratory disease in lung cancer patients and higher prevalence of diabetes in liver and pancreatic cancer patients, compared to all other cancer sites.

### 4.2 Comparison to literature

We found more than three-quarters of cancer patients had one or more pre-existing conditions. Our findings are relatively high compared to estimates of morbidity in cancer patients based on secondary care data [[10], [11], [12]], and more in line with estimates from studies that used primary care data [15,9,18]. However, direct comparisons of our findings to previous estimates are challenging, as quantifying the burden of pre-existing morbidity in cancer patients will be influenced by many factors including which chronic conditions are examined and how they are defined; whether information on conditions is self-reported by patients or derived from health records; the underlying characteristics of the study population including its age structure; and the case-mix of cancer sites included in a study.

Our findings indicate that older and male cancer patients resident in socio-economically deprived areas were more likely to have long-term conditions before cancer diagnosis. This is largely in line with previously reported morbidity patterns in both the general population and cancer patient population [3,15,4,12,21].

We reported variation in both the observed and the age-/sex-standardised prevalence of certain morbidities among 21 common and rarer cancer sites, augmenting previously observed patterns in studies that focused on a smaller range of cancers or did not standardise prevalence estimates. The findings of relatively higher prevalence of respiratory and cardiovascular disease among lung cancer patients, and higher prevalence of diabetes among liver/pancreatic cancer patients concord with prior evidence [9,10,12].

### 4.3 Strengths and limitations

Our findings are based on a nationally representative cancer patient population, identified from cancer registration and validated through primary care records. Estimating the prevalence of pre-existing chronic conditions based on clinician’s review of primary care records can provide a more complete picture of morbidities compared to studies utilising secondary care records (which may miss less severe conditions [13]) or structured primary care data (which may miss information captured in free-text format [22]). Furthermore, we included many rarer cancer sites for which there is currently limited evidence regarding pre-existing morbidities.

Several limitations merit discussion. The audit questionnaire only allowed for the recording of the presence or absence of 11 individual conditions. For example, if an individual had two distinct conditions falling into the same category (e.g. chronic back pain and arthritis), they will have been counted once, therefore potentially leading to the under-estimation of the number of conditions present. Accordingly, the median number of morbidities we report is difficult to compare externally with previous research, although it remains useful for comparing the observed number of morbidities by patient characteristics in the study population.

The validity of the information is dependent on the completeness and accuracy of primary care records, and its interpretation by primary care professionals while completing the audit; we were unable to examine the completeness of information as captured by the NCDA. Nevertheless, the studied morbidities included common chronic conditions, and are likely to have been well-recorded as they mostly reflect those covered by the Quality and Outcomes Framework (QOF), a pay-for-performance scheme that rewards practices for recording and managing certain conditions. There is no evidence to suggest that morbidities would be differentially recorded among individuals subsequently diagnosed with cancer, compared to other registered individuals in primary care practices.

### 4.4 Implications

Variation in morbidity prevalence by cancer site may reflect shared risk factors between certain morbidities and cancers. For example, smoking is a risk factor for both chronic respiratory illness and lung cancer, leading to much higher prevalence of respiratory illness in lung cancer patients compared to those with other cancers. These findings highlight the importance of public health strategies that encompass the prevention of cancer and other chronic diseases [23].

Our findings indicate that the majority of cancer patients have pre-existing conditions, commonly including cardio-metabolic, respiratory, and musculoskeletal disease. One in two patients were living with multiple conditions prior to cancer diagnosis, and 11 % had been previously diagnosed with cancer. Pre-existing morbidities can influence the processes of symptom appraisal and help-seeking behaviour by patients, and decision-making by doctors regarding referrals and investigations [8]. This could be associated with both shorter and longer intervals to diagnosis and treatment, and merits further examination through mixed-methods approaches [24]. Additionally, a new tumour could aggravate previously sub-clinical morbidities, or else be misdiagnosed as a chronic condition [16,25,26]. Further research is needed to untangle the complex associations between morbidities and cancer diagnosis.

Further, morbidities and multi-morbidities are important factors that need to be considered throughout the clinical management of cancer (encompassing treatment decisions, rehabilitation, and survivorship) [27,28]. Given that the influence of morbidity on diagnostic investigations and treatment outcomes in cancer patients are likely to be both morbidity- and cancer site-specific, the findings can guide such inquiries in future research.

## Ethics

Ethical approval was obtained by the London Hampstead Research Ethics Committee (REC reference: 20/EE/0103).

## CRediT authorship contribution statement

**Minjoung Monica Koo:** Conceptualization, Methodology, Formal analysis, Writing - original draft. **Ruth Swann:** Data curation, Writing - review & editing. **Sean McPhail:** Data curation, Writing - review & editing. **Gary A. Abel:** Methodology, Writing - review & editing. **Cristina Renzi:** Methodology, Writing - review & editing, Supervision. **Greg P. Rubin:** Conceptualization, Writing - review & editing. **Georgios Lyratzopoulos:** Methodology, Conceptualization, Writing - review & editing, Supervision.

## Declaration of Competing Interest

The authors declare no conflict of interest.
