# Supplementary material for: The prevalence of chronic conditions in patients diagnosed with one of 29 common and rarer cancers: A cross-sectional study using primary care data
Source: Cancer Epidemiol. 2020 Dec;69:101845. doi: 10.1016/j.canep.2020.101845 (PMC7768190; doi:10.1016/j.canep.2020.101845)
Supplement: Supplementary file 1 [file mmc1.docx]

Supplementary material to:

Koo MM, Swann R, Mcphail S, et al., The prevalence of chronic conditions in patients diagnosed with one of 29 common and rarer cancers: A cross-sectional study using primary care data

Cancer Epidemiology 2020

## Table of contents

- Supplementary material I: Comparison of study population and standard population
- Supplementary material II: Observed prevalence of morbidity by cancer type
- Supplementary material III: Standardised prevalence of morbidity by cancer type
- Supplementary material IV: Prevalence of morbidity by cancer (visualised by cancer)

### Supplementary material I: Comparison of the study population and standard population (used for direct standardisation of prevalence estimates)

Sex- and age-specific mid-year estimates of the English population in 2014 were used as the standard population when producing directly standardised prevalence estimates of morbidities among the NCDA study population (Office for National Statistics, 2016a).

The standard population was younger than the NCDA study population, with slightly greater differences among men than women.

|  | NCDA study population | | Standard population | |
| --- | --- | --- | --- | --- |
|  | N | % of pop | N | % of pop |
| Males |  |  |  |  |
| 35-44 years | 208 | 3% | 3,533,203 | 24% |
| 45–54 years | 589 | 7% | 3,775,061 | 26% |
| 55–64 years | 1,481 | 19% | 3,002,233 | 21% |
| 65–74 years | 2,599 | 33% | 2,487,171 | 17% |
| 75–84 years | 2,173 | 28% | 1,380,943 | 9% |
| 85+ years | 833 | 11% | 438,564 | 3% |
| Total | 7,883 | 100% | 14,617,175 | 100% |
| Females |  |  |  |  |
| 35-44 years | 208 | 3% | 3,570,205 | 23% |
| 45–54 years | 589 | 7% | 3,860,590 | 24% |
| 55–64 years | 1,481 | 19% | 3,098,279 | 20% |
| 65–74 years | 2,599 | 33% | 2,675,702 | 17% |
| 75–84 years | 2,173 | 28% | 1,718,376 | 11% |
| 85+ years | 833 | 11% | 836,952 | 5% |
| Total | 7,883 | 100% | 15,760,104 | 100% |

### Supplementary material II: Observed prevalence of morbidity by cancer type

NB percentages are calculated in rows (e.g. 42% of prostate cancer patients had hypertension).

| Cancer site* | Hyp. | CVD | MSK | Diabetes | Resp. | Prev. canc. | CBD | Cog. Imp. | SMI | Phys. Dis. | Other |
| --- | --- | --- | --- | --- | --- | --- | --- | --- | --- | --- | --- |
| Prostate | 42% (39–44%) | 23% (21–24%) | 19% (17–21%) | 15% (14–17%) | 12% (11–14%) | 6% (6–8%) | 6% (5–7%) | 3% (2–4%) | 1% (1–2%) | 1% (1–2%) | 19% (18–21%) |
| Lung | 39% (37–41%) | 28% (26–30%) | 21% (19–22%) | 16% (14–17%) | 35% (33–37%) | 13% (12–14%) | 10% (9–12%) | 6% (5–7%) | 4% (3–5%) | 2% (2–3%) | 20% (19–22%) |
| Breast | 33% (31–36%) | 13% (11–14%) | 19% (17–21%) | 11% (9–12%) | 10% (9–12%) | 9% (8–11%) | 5% (4–6%) | 5% (4–6%) | 3% (2–4%) | 1% (1–2%) | 18% (16–20%) |
| Colon | 42% (39–45%) | 23% (21–26%) | 17% (15–19%) | 18% (15–20%) | 13% (11–15%) | 13% (11–15%) | 9% (8–11%) | 6% (5–7%) | 2% (1–3%) | 2% (1–2%) | 23% (20–25%) |
| Melanoma | 34% (31–37%) | 15% (12–18%) | 16% (14–19%) | 11% (9–14%) | 9% (7–11%) | 14% (12–17%) | 5% (4–7%) | 4% (3–6%) | 1% (1–2%) | 0.7% (0.3–1.5%) | 17% (15–20%) |
| Lymphoma | 35% (31–38%) | 23% (20–26%) | 19% (16–22%) | 14% (12–17%) | 14% (11–17%) | 13% (11–16%) | 6% (4–8%) | 2% (2–4%) | 3% (2–4%) | 2% (1–4%) | 21% (18–24%) |
| Other | 38% (34–42%) | 20% (17–23%) | 15% (12–18%) | 16% (13–19%) | 12% (10–15%) | 14% (12–17%) | 6% (5–8%) | 5% (4–7%) | 2% (1–4%) | 2% (1–4%) | 19% (16–22%) |
| Rectal | 41% (37–45%) | 23% (20–27%) | 17% (14–20%) | 19% (16–23%) | 11% (9–14%) | 8% (6–11%) | 7% (6–10%) | 3% (2–5%) | 2% (1–3%) | 1.1% (0.5–2.3%) | 18% (15–22%) |
| Renal | 49% (44–53%) | 25% (21–29%) | 18% (15–21%) | 19% (16–22%) | 13% (10–16%) | 14% (11–17%) | 10% (7–12%) | 5% (3–7%) | 3% (2–5%) | 2% (1–3%) | 19% (16–22%) |
| Bladder | 44% (39–48%) | 29% (25–33%) | 19% (16–23%) | 18% (15–22%) | 12% (9–15%) | 14% (11–18%) | 10% (7–13%) | 7% (5–10%) | 3% (2–5%) | 1% (1–3%) | 22% (18–26%) |
| Pancreatic | 37% (32–41%) | 21% (18–25%) | 17% (14–21%) | 28% (24–33%) | 12% (9–15%) | 14% (11–17%) | 8% (6–10%) | 5% (4–8%) | 2% (1–4%) | 2% (1–4%) | 20% (17–24%) |
| Leukaemia | 33% (28–38%) | 20% (16–24%) | 16% (13–20%) | 14% (11–17%) | 12% (9–16%) | 16% (13–20%) | 5% (3–8%) | 4% (2–6%) | 3% (2–5%) | 1.0% (0.4–2.6%) | 20% (17–25%) |
| Oesophageal | 40% (35–45%) | 25% (21–29%) | 17% (14–21%) | 18% (15–22%) | 19% (15–23%) | 12% (9–16%) | 9% (7–13%) | 4% (3–6%) | 3% (2–5%) | 0% | 24% (20–28%) |
| Endometrial | 46% (41–51%) | 12% (10–16%) | 22% (18–27%) | 22% (18–26%) | 8% (6–11%) | 10% (7–13%) | 4% (2–6%) | 4% (2–6%) | 3% (1–5%) | 2% (1–4%) | 18% (15–22%) |
| CUP | 46% (42–51%) | 28% (24–33%) | 23% (19–28%) | 18% (14–22%) | 18% (14–22%) | 13% (10–17%) | 11% (8–15%) | 8% (5–11%) | 2% (1–4%) | 4% (2–6%) | 20% (17–25%) |
| Ovarian | 34% (29–39%) | 12% (9–16%) | 18% (14–23%) | 12% (9–16%) | 9% (6–12%) | 9% (6–12%) | 4% (3–7%) | 2% (1–4%) | 3% (1–5%) | 0.6% (0.2–2.3%) | 17% (13–21%) |
| Stomach | 43% (38–49%) | 24% (19–29%) | 17% (13–22%) | 20% (16–25%) | 17% (13–22%) | 11% (8–15%) | 8% (5–11%) | 6% (4–9%) | 3% (2–6%) | 2% (1–5%) | 19% (15–24%) |
| Oral/ oropharyngeal | 34% (28–39%) | 16% (12–20%) | 13% (9–17%) | 13% (9–17%) | 14% (11–19%) | 6% (4–10%) | 6% (4–9%) | 4% (2–7%) | 4% (2–7%) | 1.0% (0.4–3.0%) | 25% (20–30%) |
| Myeloma | 42% (37–48%) | 22% (17–27%) | 24% (20–30%) | 16% (12–21%) | 12% (8–16%) | 8% (5–12%) | 7% (4–11%) | 5% (3–8%) | 2% (1–5%) | 2% (1–5%) | 27% (22–32%) |
| Liver | 46% (40–52%) | 27% (22–33%) | 22% (17–27%) | 38% (33–44%) | 14% (11–19%) | 11% (8–15%) | 5% (3–8%) | 4% (2–7%) | 2% (1–4%) | 5% (3–8%) | 37% (31–43%) |
| Brain/CNS | 28% (23–35%) | 16% (12–22%) | 10% (7–15%) | 15% (10–20%) | 10% (7–15%) | 13% (9–18%) | 6% (4–10%) | 4% (2–7%) | 4% (2–8%) | 0.9% (0.3–3.4%) | 14% (10–19%) |

Hyp=hypertension; CVD=cardiovascular disease; MSK=musculo-skeletal; CBD: cerebrovascular disease; Resp=respiratory; Prev. canc.=previous cancer; Cog. Imp=cognitive impairment; SMI=severe mental illness; Phys. Dis.=physical disability

*Cancers for which n<200 patients were excluded (thyroid, mesothelioma, laryngeal, small intestine, cervical, vulval, gallbladder, and testicular cancers)

### Supplementary material III: Standardised prevalence of morbidity by cancer type

NB percentages are calculated in rows (e.g. 24% of prostate cancer patients had hypertension).

| Cancer site* | Hyp. | CVD | MSK | Diabetes | Resp. | Prev. canc. | CBD | Cog. Imp. | SMI | Phys. Dis. | Other |
| --- | --- | --- | --- | --- | --- | --- | --- | --- | --- | --- | --- |
| Prostate | 26% (24–28%) | 11% (9–12%) | 12% (10–13%) | 9% (8–10%) | 13% (12–15%) | 3% (3–4%) | 3% (2–4%) | 1% (1–2%) | 1% (1–2%) | 1% (1–1%) | 13% (12–15%) |
| Lung | 24% (22–26%) | 13% (12–15%) | 11% (10–13%) | 8% (7–10%) | 24% (22–26%) | 7% (6–9%) | 5% (4–6%) | 2% (2–3%) | 4% (3–5%) | 1% (1–2%) | 21% (19–22%) |
| Breast | 24% (22–26%) | 8% (7–9%) | 15% (13–16%) | 8% (7–10%) | 9% (8–10%) | 8% (7–9%) | 3% (2–4%) | 3% (2–4%) | 3% (2–4%) | 1% (1–2%) | 16% (15–18%) |
| Colon | 24% (21–26%) | 11% (10–13%) | 10% (9–12%) | 11% (10–13%) | 10% (8–11%) | 7% (6–9%) | 4% (3–6%) | 3% (2–4%) | 2% (1–3%) | 1% (1–2%) | 22% (20–25%) |
| Melanoma | 25% (22–28%) | 8% (6–10%) | 12% (9–14%) | 9% (7–11%) | 8% (6–10%) | 9% (8–12%) | 3% (2–4%) | 3% (2–4%) | 1% (1–2%) | 0.3% (0.1–1.0%) | 14% (12–17%) |
| Lymphoma | 20% (18–24%) | 12% (10–15%) | 13% (10–15%) | 10% (8–12%) | 10% (8–13%) | 8% (6–10%) | 3% (2–4%) | 1% (1–3%) | 3% (2–5%) | 3% (2–4%) | 22% (19–25%) |
| Other | 25% (22–29%) | 12% (9–14%) | 12% (10–15%) | 12% (10–15%) | 10% (7–12%) | 9% (7–12%) | 3% (2–5%) | 3% (2–4%) | 2% (1–4%) | 1% (1–3%) | 20% (17–23%) |
| Rectal | 24% (21–28%) | 11% (8–14%) | 12% (10–15%) | 13% (10–16%) | 7% (5–10%) | 5% (3–7%) | 3% (2–5%) | 1% (1–3%) | 1% (1–2%) | 1.0% (0.4–2.2%) | 15% (12–18%) |
| Renal | 32% (28–36%) | 14% (12–18%) | 13% (11–17%) | 14% (12–18%) | 11% (8–14%) | 12% (10–15%) | 7% (5–9%) | 3% (2–5%) | 3% (2–5%) | 1.0% (0.4–2.3%) | 17% (14–21%) |
| Bladder | 27% (23–31%) | 14% (11–17%) | 11% (9–14%) | 8% (6–11%) | 6% (4–9%) | 10% (8–13%) | 4% (2–6%) | 3% (2–5%) | 9% (7–12%) | 3% (2–5%) | 22% (19–26%) |
| Pancreatic | 21% (18–25%) | 8% (6–11%) | 8% (6–11%) | 20% (16–23%) | 8% (5–10%) | 11% (9–14%) | 3% (2–5%) | 2% (1–3%) | 2% (1–4%) | 1.0% (0.4–2.4%) | 22% (19–27%) |
| Leukaemia | 17% (13–21%) | 10% (7–13%) | 11% (8–15%) | 11% (8–14%) | 11% (8–15%) | 12% (9–16%) | 3% (2–6%) | 2% (1–4%) | 4% (3–7%) | 1% (1–3%) | 20% (16–24%) |
| Oesophageal | 20% (17–24%) | 12% (9–15%) | 9% (6–12%) | 13% (10–16%) | 13% (10–17%) | 18% (15–22%) | 5% (3–7%) | 4% (3–6%) | 2% (1–4%) | 0% | 25% (21–29%) |
| Endometrial | 35% (31–40%) | 8% (6–12%) | 15% (12–19%) | 20% (16–24%) | 10% (7–13%) | 6% (4–9%) | 2% (1–4%) | 3% (1–5%) | 3% (2–5%) | 2% (1–4%) | 22% (18–26%) |
| CUP | 24% (20–28%) | 12% (9–16%) | 14% (11–18%) | 10% (7–13%) | 16% (13–20%) | 7% (5–10%) | 5% (3–7%) | 2% (1–4%) | 3% (2–6%) | 2% (1–4%) | 22% (19–27%) |
| Ovarian | 23% (18–27%) | 7% (5–11%) | 14% (10–18%) | 9% (6–13%) | 7% (5–10%) | 6% (4–9%) | 3% (1–5%) | 1.0% (0.4–2.9%) | 4% (3–7%) | 0.4% (0.1–1.8%) | 18% (14–22%) |
| Stomach | 22% (18–27%) | 13% (9–17%) | 8% (6–12%) | 10% (7–14%) | 9% (6–12%) | 6% (4–9%) | 3% (2–6%) | 2% (1–4%) | 3% (2–6%) | 1% (1–3%) | 12% (9–16%) |
| Oral/ oropharyngeal | 24% (20–29%) | 11% (8–15%) | 12% (8–16%) | 9% (6–12%) | 11% (8–16%) | 4% (2–7%) | 4% (3–7%) | 3% (1–5%) | 6% (4–10%) | 2% (1–5%) | 21% (17–26%) |
| Myeloma | 27% (22–33%) | 12% (9–17%) | 17% (13–22%) | 10% (7–15%) | 16% (12–21%) | 6% (4–9%) | 4% (2–7%) | 2% (1–4%) | 0.8% (0.2–2.8%) | 0.8% (0.2–2.8%) | 25% (20–30%) |
| Liver | 27% (22–33%) | 11% (7–15%) | 14% (10–19%) | 21% (16–26%) | 11% (8–16%) | 5% (3–9%) | 2% (1–4%) | 3% (2–7%) | 4% (2–7%) | 4% (3–8%) | 46% (40–52%) |
| Brain/CNS | 19% (15–25%) | 9% (6–13%) | 7% (4–11%) | 9% (6–14%) | 7% (4–11%) | 9% (6–14%) | 5% (2–8%) | 3% (1–6%) | 4% (2–8%) | 0.4% (0.1–2.5%) | 13% (9–18%) |

Hyp=hypertension; CVD=cardiovascular disease; MSK=musculo-skeletal; CBD: cerebrovascular disease; Resp=respiratory; Prev. canc.=previous cancer; Cog. Imp=cognitive impairment; SMI=severe mental illness; Phys. Dis.=physical disability

* Cancers for which n<200 patients were excluded (thyroid, mesothelioma, laryngeal, small intestine, cervical, vulval, gallbladder, and testicular cancers)

### Supplementary material IV: Prevalence of morbidity by cancer (visualised by cancer)

Observed and standardised prevalence of morbidity, visualised by cancer site for the ten most common cancers. The x-axis is ordered by standardised prevalence of eleven morbidities, in increasing order. See Table S2-3 for underlying prevalence estimates and Figure 4 (main text) for the same estimates visualised by morbidity.


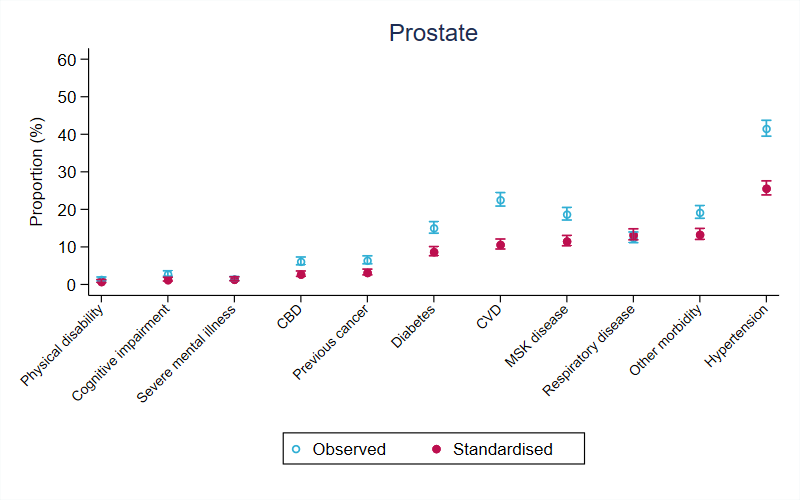


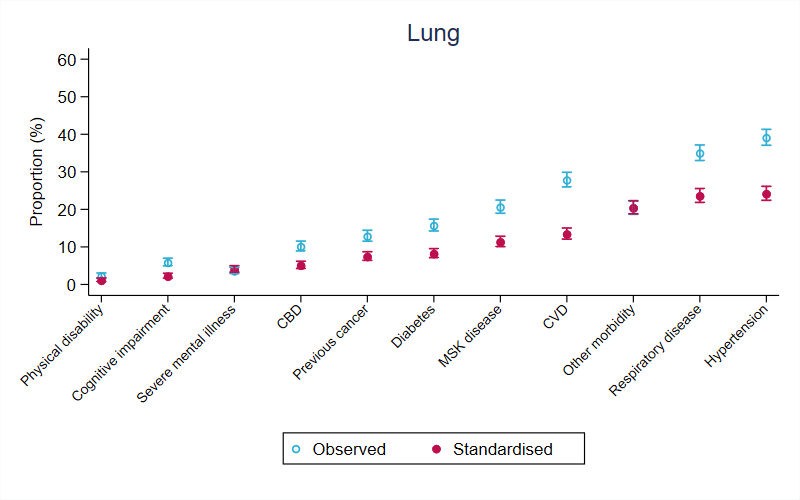


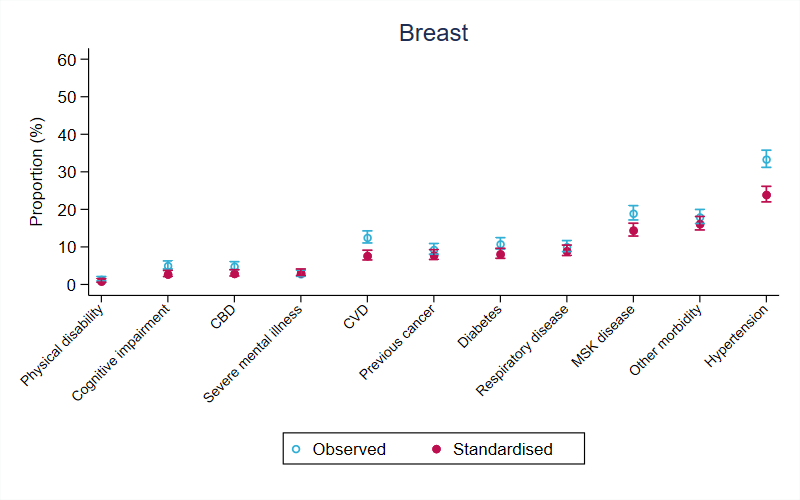


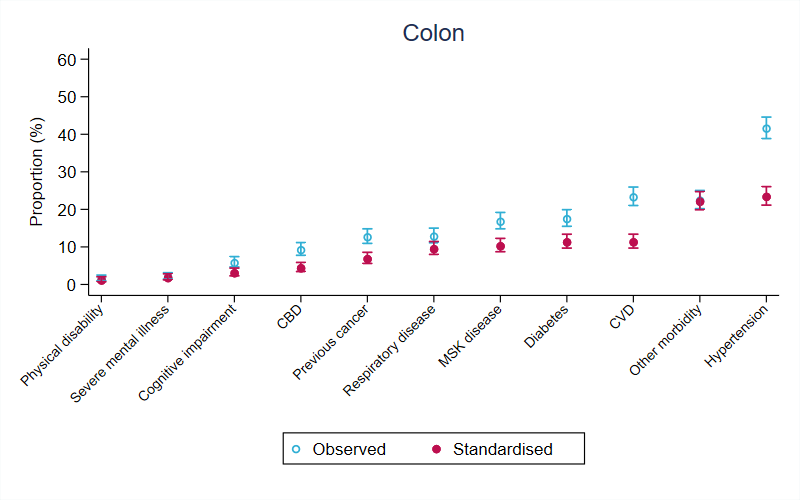


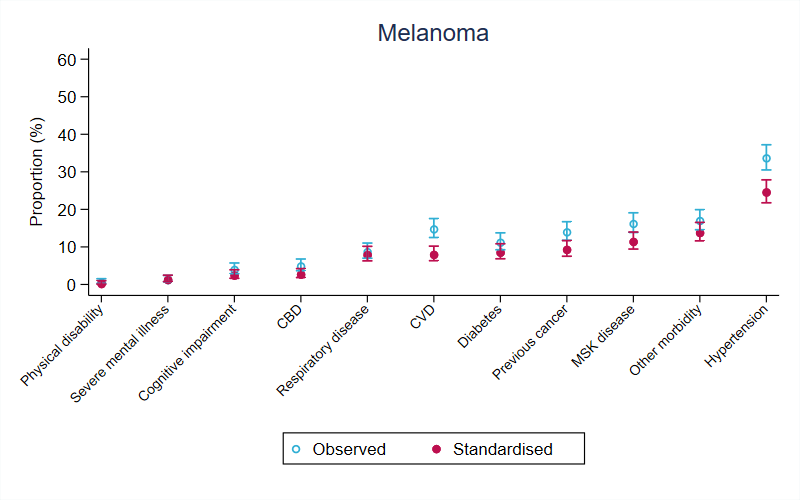


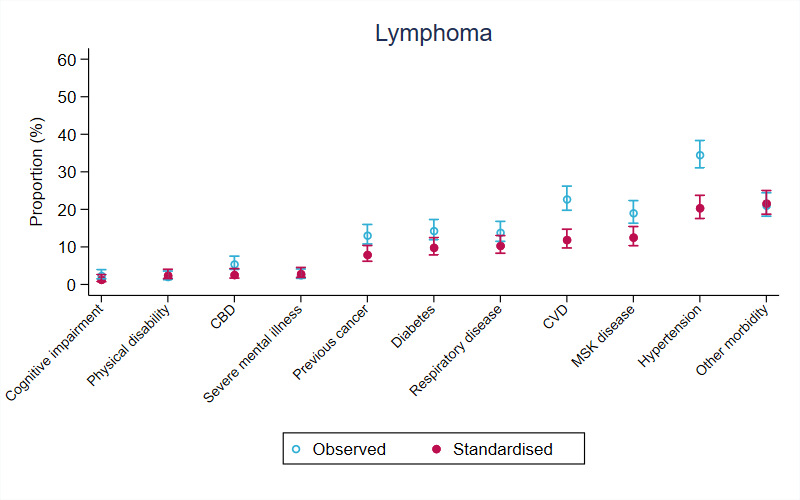


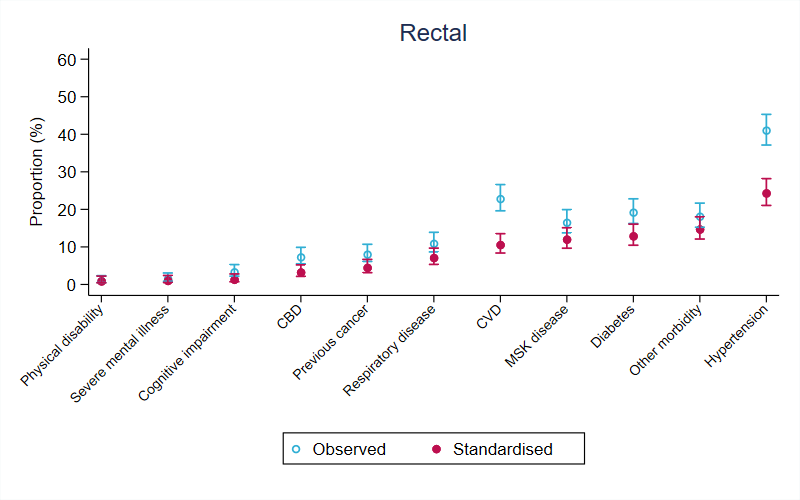


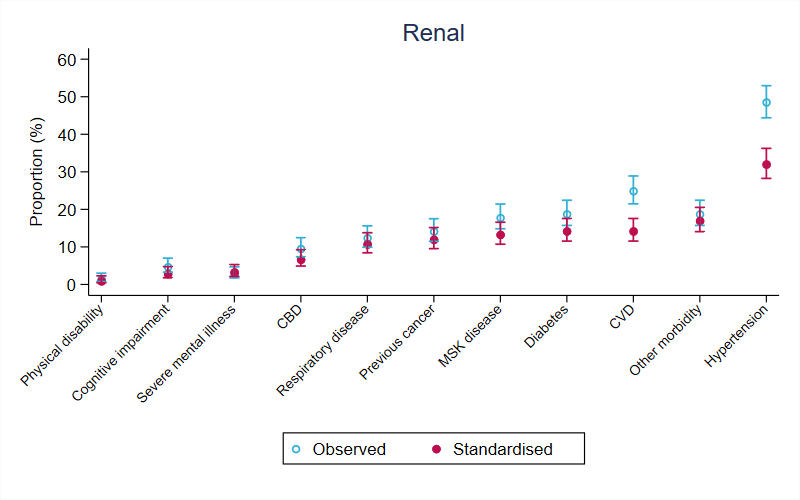


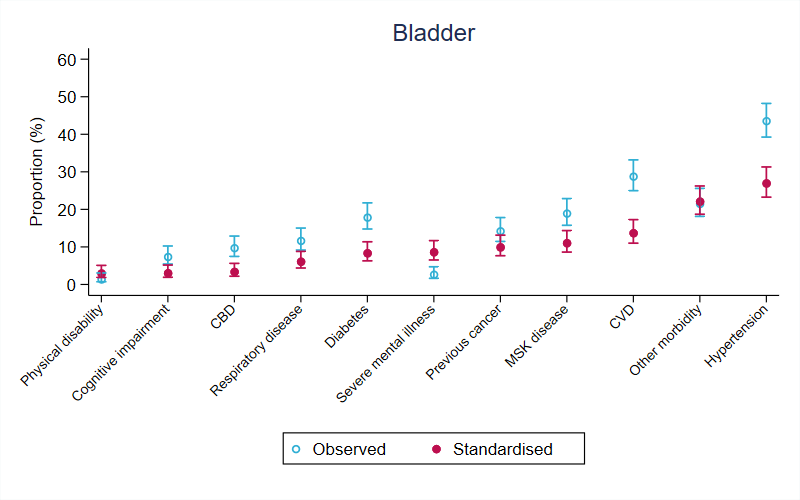


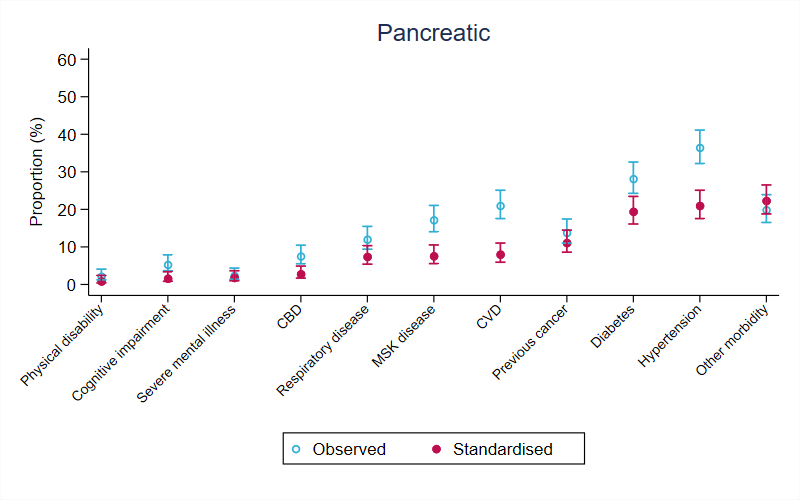


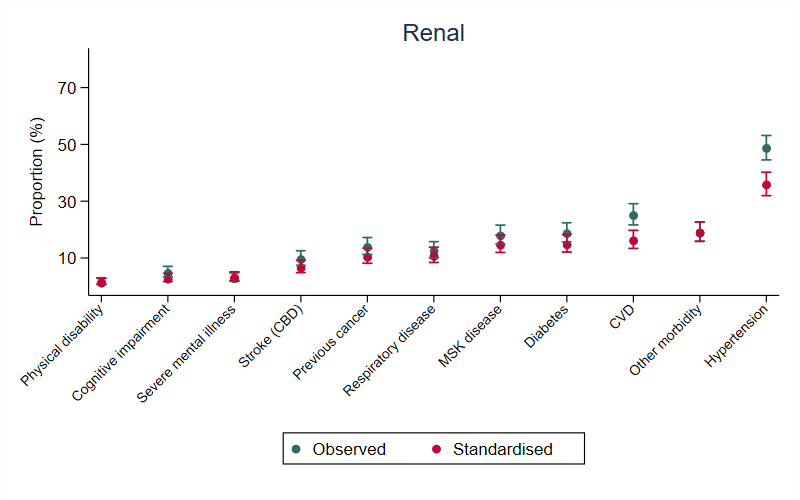
[End of supplementary material]
